# Supplementary material for: Rhizobia independently adapt to soil and legume host environments, but soil conditions influence the abundance of high-quality partners
Source: mBio. 2026 May 11;17(6):e03793-25. doi: 10.1128/mbio.03793-25 (PMC13251399; doi:10.1128/mbio.03793-25)

Supplemental information for:

Title: Rhizobia independently adapt to soil and legume host environments, but soil conditions influence the abundance of high-quality partners.

Alejandra Gil-Polo,[a] Regina B. Bledsoe,[a] McCall Calvert,[b]* Lillian Cherry,[a] Brendan Epstein,[d] Rebecca Fudge,[c]* Jennifer Harris,[a] Peter Tiffin,[d] Liana T. Burghardt[a]#

[a]*Department of Plant Science, The Pennsylvania State University, University Park, USA;*
[b]*Department of Biology, University of Pennsylvania, Philadelphia, Pennsylvania, USA;*
[c]*Department of Horticultural Science, University of Minnesota, Saint Paul, Minnesota, USA;*
[d]*Department of Plant and Microbial Biology, University of Minnesota, Saint Paul, Minnesota, USA.*

Table S1: The abiotic treatments (Trt) affect the population size across timepoints (Time). The population size is represented as $\log_{10}$(CFU/mesocosm). (A) Two-way ANOVA Full Model. (B) Dunnett's *post hoc* test compared population size in field soil control (FS-control, also referred to as FS) with each soil perturbation (listed under Comparison) within each timepoint. Df (Degrees of freedom), model sum of squares (Sum sq), *P* (P-value), F value (F-statistics), Estimate (Estimated differences in means).

(A)

**Term**	**Df**	**Sum Sq**	**F value**	***P***
Time	3	70.310	1886.231	**< 0.001**
Trt	7	15.672	180.183	**< 0.001**
Time:Trt	20	14.900	59.958	**< 0.001**
Residuals	113	1.404	NA	

(B)

**Comparison**	**2 wk**		**10 wk**		**24 wk**	
	**Estimate**	***P***	**Estimate**	***P***	**Estimate**	***P***
32°C - FS	0.054	0.972	-0.446	**< 0.001**	-0.262	**0.004**
4°C - FS	-2.093	**< 0.001**	-1.459	**< 0.001**	-0.385	**< 0.001**
Na+ - FS	0.223	*0.067*	0.143	0.367	-0.458	**< 0.001**
La - FS	-0.124	0.518	-0.247	**0.028**	-0.179	**0.010**
Sm - FS	0.118	0.566	0.042	0.995	-0.090	0.388
Clay - FS	0.136	0.429	-0.161	0.257	-0.419	**< 0.001**
Dry - FS			-0.071	0.917	-0.347	**< 0.001**

Table S2: Temperature treatment has a significant effect on the rhizobial diversity at later timepoints. (A) Two-way ANOVA, full model. (B) Dunnett's *post hoc* test on Shannon diversity comparing the field soil control (FS) to all the other abiotic treatments (Comparison) within each timepoint. We were unable to calculate the frequency of rhizobial strains at 4 °C at the 2-week time point due to a low DNA yield. We also did not calculate Dry at 2 weeks because the tubes took about 4 weeks to dry out. Comparison (Treatments contrasted), Df (Degrees of freedom), Sum sq (model sum of squares), *P* (P-value), F value (F-statistics), Estimate (Estimated differences in means).

(A)

**Term**	**Df**	**Sum Sq**	**F value**	***P***
Trt	7	2.429	84.630	**< 0.001**
Time	2	0.107	13.065	**< 0.001**
Trt:Time	12	0.289	5.874	**< 0.001**
Residuals	80	0.328	NA	

(B)

**Comparison**	**2wk**		**10wk**		**24wk**	
	**Estimate**	***P***	**Estimate**	***P***	**Estimate**	***P***
32°C - FS	-0.067	*0.052*	-0.332	**< 0.001**	-0.376	**< 0.001**
Clay - FS	0.019	0.914	-0.073	0.517	-0.074	0.433
La - FS	0.046	0.393	0.027	0.989	0.008	1.000
Na+ - FS	0.026	0.745	0.028	0.985	0.036	0.942
Sm - FS	-0.069	**0.043**	-0.088	0.274	-0.093	0.259
4°C - FS			-0.404	**< 0.001**	-0.625	**< 0.001**
Dry - FS			-0.017	0.999	-0.059	0.712

Table S3: Redundancy analysis reveals a significant interaction of time and treatment (Trt) in the rhizobial population composition in the soil. (A) Variance partitioning of rhizobial strain fitness explained by Time and Trt. The component “Constrained” represents the variance explained by the explanatory variables Time and Treatment, whereas the “Unconstrained” component is the variance not explained by those explanatory variables. The column “Inertia” represents the total variance of the data explained by the constrained or unconstrained component while the “Proportion” column represents the proportion of the total variation explained by both components. (B) Permutation test results for the Full RDA model. Df (Degrees of freedom), *P* (P-value), Prop.Var (Proportion of Variance), F value (F-statistics), R^2adj (Adjusted R-squared).

(A)

**Component**	**Inertia**	**Proportion**
Total	68	1
Constrained	61.257	0.901
Unconstrained	6.743	0.11

(B)

**Term**	**Df**	**Prop.Var**	**F value**	***P***
**R^2adj =**	0.875			
Trt	7	0.779	89.76	**0.001**
Time	2	0.043	17.2	**0.001**
Trt:Time	12	0.079	5.33	**0.001**
Residual	80	0.099		

Table S4: Cold, salinity, and warm treatments explained the strongest population shifts. Pairwise comparisons were performed using a permutation test on RDA models that included Treatment and Time. Each comparison represents the contrast between the field soil control (FS) vs other soil treatments. Comparison (Treatments contrasted), R^2.adj (Adjusted R-squared) Df (Degrees of freedom), *P* (P-value), F value (F-statistics).

**Comparison**	**R^2.adj**	**Term**	**Df**	**F value**	***P***
4°C - FS	0.872	**Trt**	1	120.06	**0.001**
		**Time**	2	12.91	**0.001**
		**Trt:Time**	1	14.37	**0.001**
		Residual	19		
Na+ - FS	0.704	**Trt**	1	57.3	**0.001**
		**Time**	2	5.36	**0.002**
		**Trt:Time**	2	3.06	**0.025**
		Residual	24		
32°C - FS	0.609	**Trt**	1	30.2	**0.001**
		**Time**	2	4.65	**0.001**
		**Trt:Time**	2	4.56	**0.002**
		Residual	23		
Clay - FS	0.584	**Trt**	1	20.31	**0.001**
		**Time**	2	8.35	**0.001**
		**Trt:Time**	2	3.64	**0.003**
		Residual	23		
La - FS	0.469	**Trt**	1	11.72	**0.001**
		**Time**	2	6.79	**0.001**
		Trt:Time	2	1.34	0.199
		Residual	21		
Dry - FS	0.443	**Trt**	1	6.47	**0.001**
		**Time**	2	6.43	**0.001**
		**Trt:Time**	1	2.94	**0.011**
		Residual	19		
Sm - FS	0.443	**Trt**	1	11.39	**0.001**
		**Time**	2	6.9	**0.001**
		Trt:Time	2	1.05	0.403
		Residual	23		

Table S5: Shannon diversity in the field soil control (FS) is influenced by time. (A) Full ANOVA model. (B) Tukey HSD *post-hoc* test used to compare diversity across timepoints (Comparison). Df (Degrees of freedom), Sum sq (model sum of squares), *P* (P-value), F value (F-statistics), Diff (Difference between the means of two groups)

(A)

**Term**	**Df**	**Sum Sq**	**F value**	***P***
Time	3	0.071	41.545	**< 0.001**
Residuals	16	0.009	NA	

(B)

**Comparison**	**Diff**	***P***
2wk-10wk	-0.025	0.376
2wk-24wk	-0.013	0.839
2wk-2.5y	0.124	**< 0.001**
10wk-24wk	-0.012	0.841
10wk-2.5y	-0.149	**< 0.001**
24wk-2.5y	0.136	**< 0.001**

Table S6: Redundancy analysis reveals a significant effect of time on the rhizobial population composition in the field soil (FS-control). (A) Variance partitioning of rhizobial strain fitness explained by Time. Shown are the total variance (Inertia) and the proportion of variance (Proportion) explained by the constrained component (Time), and the unconstrained (residual) component. (B) Permutation test results for the RDA model. Df (Degrees of freedom), *P* (P-value), Prop.Var (Proportion of Variance), F value (F-statistics), R^2adj (Adjusted R-squared).

(A)

**Component**	**Inertia**	**Proportion**
Total	68	1
Constrained	41.699	0.613
Unconstrained	26.301	0.387

(B)

**Term**	**Df**	**Prop.Var**	**F value**	***P***
**R2.adj =**	0.541			
Time	3	0.613	8.456	**0.001**
Residual	16	0.387		

Table S7: Pearson correlation coefficients (R) show that the rhizobial relative fitness at 24 weeks is positively correlated across soil treatments. Comparison (Treatments contrasted), *P* (P-value).

**Comparison**	**R**	***P***
FS vs 4°C	0.396	**0.001**
FS vs 32°C	0.723	**<0.001**
FS vs Na+	0.563	**<0.001**
FS vs Clay	0.883	**<0.001**
FS vs Sm	0.958	**<0.001**
FS vs La	0.949	**<0.001**
FS vs Dry	0.959	**<0.001**
4°C vs 32°C	0.365	**0.002**
4°C vs Na+	0.367	**0.002**
4°C vs Clay	0.387	**0.001**
4°C vs Sm	0.444	**<0.001**
4°C vs La	0.455	**<0.001**
4°C vs Dry	0.437	**<0.001**
32°C vs Na+	0.591	**<0.001**
32°C vs Clay	0.558	**<0.001**
32°C vs Sm	0.791	**<0.001**
32°C vs La	0.671	**<0.001**
32°C vs Dry	0.707	**<0.001**
Na+ vs Clay	0.553	**<0.001**
Na+ vs Sm	0.644	**<0.001**
Na+ vs La	0.442	**<0.001**
Clay vs Dry	0.897	**<0.001**
Clay vs Sm	0.865	**<0.001**
Clay vs La	0.841	**<0.001**
Clay vs Dry	0.897	**<0.001**
Sm vs La	0.895	**<0.001**
Sm vs Dry	0.950	**<0.001**
La vs Dry	0.932	**<0.001**

Table S8: Pearson correlation coefficients (R) show no significant correlations between rhizobial strain relative fitness in the soil at 24 weeks and rhizobial strain relative fitness in nodules of the hosts *Medicago truncatula* A17 or *M. littoralis* R108. Comparison (Treatments contrasted), *P* (P-value).

**Comparison**	**R**	***P***
A17 - FS	0.083	0.501
A17 - 4°C	0.166	0.175
A17 - 32°C	0.112	0.364
A17 - Na+	0.221	*0.070*
A17 - Clay	0.162	0.188
A17 - Sm	0.125	0.308
A17 - La	0.064	0.602
A17 - Dry	0.046	0.707
R108 - FS	0.121	0.326
R108 - 4°C	0.059	0.634
R108 - 32°C	0.103	0.404
R108 - Na+	0.219	*0.073*
R108 - Clay	0.181	0.140
R108 - Sm	0.118	0.337
R108 - La	0.086	0.484
R108 - Dry	0.072	0.559

Table S9: The LD groups that crossed the Bonferroni threshold in the Genome-Wide Association study were limited to three soil abiotic perturbations. The Bonferroni threshold differed per replicon because the GWA study was run independently in each replicon: Chromosome ($1.718*10^{-05}$), pSymA ($4.036*10^{-06}$), and pSymB ($2.832*10^{-06}$). Replicon (Replicon where the LD group is located), Beta (allelic effect size), *P* (P-value, significance), Group size (LD group size that shows the number of genes in each group), Locus tag (NCBI *S. meliloti* USDA1106), Product (putative product).

**Trt**	**Replicon**	**Beta**	***P***	**Group size**	**Locus_tag**	**Product**
32°C	pSymB	0.915	$2.75*10^{-06}$	1	CDO30_RS28455	FAD-dependent oxidoreductase; protein_id=WP_015456630.1
4°C	pSymA	1.34	$1.39*10^{-06}$	1	CDO30_RS21590	PAS domain S-box protein; protein_id=WP_013845133.1
Clay	pSymA	0.760	$1.4*10^{-06}$	1	CDO30_RS19900	hypothetical protein; protein_id=WP_010967343.1
4°C	pSymA	1.447	$2.35*10^{-06}$	1	CDO30_RS21920	repA, plasmid partitioning protein RepA; protein_id=WP_010968237.1
4°C	pSymA	1.217	$1.61*10^{-06}$	1	CDO30_RS21920	repA, plasmid partitioning protein RepA; protein_id=WP_010968237.1
4°C	pSymA	1.573	$1.74*10^{-06}$	1	CDO30_RS21395	mechanosensitive ion channel family protein; protein_id=WP_088239352.1

Table S10: Pearson correlation coefficients (R) show that there are only weak correlations between rhizobial fitness in the soil at 24 weeks and the rhizobial plant benefit to A17 (weight_A17) and R108 (weight_R108). Comparison (Treatments contrasted), *P* (P-value).

**Comparison**	**R**	***P***
weight_A17 vs FS	0.029	0.828
weight_A17 vs 4°C	0.320	**0.013**
weight_A17 vs 32°C	0.210	0.111
weight_A17 vs Na+	0.191	0.147
weight_A17 vs Clay	0.053	0.688
weight_A17 vs Sm	0.107	0.419
weight_A17 vs La	-0.001	0.996
weight_A17 vs Dry	0.056	0.676
weight_R108 vs FS	0.207	0.211
weight_R108 vs 4°C	-0.145	0.385
weight_R108 vs 32°C	0.302	*0.066*
weight_R108 vs Na+	0.258	0.118
weight_R108 vs Clay	0.304	*0.063*
weight_R108 vs Sm	0.279	*0.090*
weight_R108 vs La	0.121	0.470
weight_R108 vs Dry	0.186	0.262

Table S11: The potential benefits of the rhizobial population for hosts differ from the initial population after selection in the soil for 24 weeks in many cases, and the strength and direction depend on the host plant (Host) and soil treatments. (A) Results from separate ANOVA models for each host genotype with treatment as the predictor and potential benefit as the outcome. (B) Results of Dunnett's *post hoc* test run on each model in (A) that specifically compares the potential benefit of the initial inoculum (Initial) vs each soil treatment. Comparison (Treatments contrasted), Df (Degrees of freedom), Sum sq (model sum of squares), *P* (P-value), F value (F-statistics), Estimate (Estimated differences in means).

(A)

**Host**	**Term**	**Df**	**Sum Sq**	**F value**	***P***
A17	Trt	8	0.043	27.359	**< 0.001**
A17	Residuals	31	0.006		
R108	Trt	8	0.044	51.355	**< 0.001**
R108	Residuals	31	0.003		

(B)

**Host**	**Comparison**	**Estimate**	***P***
A17	32°C - Initial	0.087	**< 0.001**
A17	4°C - Initial	0.114	**< 0.001**
A17	Clay - Initial	0.031	**0.018**
A17	Dry - Initial	0.033	**0.014**
A17	FS - Initial	0.022	0.143
A17	La - Initial	0.024	0.116
A17	Na+ - Initial	0.038	**0.002**
A17	Sm - Initial	0.031	**0.024**
R108	32°C - Initial	0.093	**< 0.001**
R108	4°C - Initial	-0.023	**0.020**
R108	Clay - Initial	0.072	**< 0.001**
R108	Dry - Initial	0.039	**< 0.001**
R108	FS - Initial	0.037	**< 0.001**
R108	La - Initial	0.030	**0.002**
R108	Na+ - Initial	0.032	**< 0.001**
R108	Sm - Initial	0.062	**< 0.001**

Table S12: The potential benefits of the rhizobial population for hosts differ from the population of the field soil control (FS-control) after selection in the soil for 24 weeks, and the strength and direction depend on the host plant (Host) and soil abiotic perturbations. (A) Results from separate ANOVA models for each host genotype with treatment as the predictor and potential benefit as the outcome. (B) Results of Dunnett's *post hoc* test run on each model in (A) that specifically compares the potential benefit of the FS-control (FS) vs each soil treatment. Comparison (Treatments contrasted), Df (Degrees of freedom), Sum sq (model sum of squares), *P* (P-value), F value (F-statistics), Estimate (Estimated differences in means).

(A)

**Host**	**Term**	**Df**	**Sum Sq**	**F value**	***P***
A17	Trt	7	0.035	23.013	**< 0.001**
	Residuals	28	0.006		
R108	Trt	7	0.037	44.717	**< 0.001**
	Residuals	28	0.003		

(B)

**Host**	**Comparison**	**Estimate**	***P***
A17	32°C - FS	0.066	**< 0.001**
A17	4°C - FS	0.093	**< 0.001**
A17	Clay - FS	0.009	0.891
A17	Dry - FS	0.012	0.768
A17	La - FS	0.002	1.000
A17	Na+ - FS	0.017	0.358
A17	Sm - FS	0.009	0.899
R108	32°C - FS	0.056	**< 0.001**
R108	4°C - FS	-0.060	**< 0.001**
R108	Clay - FS	0.035	**< 0.001**
R108	Dry - FS	0.002	1.000
R108	La - FS	-0.006	0.918
R108	Na+ - FS	-0.004	0.984
R108	Sm - FS	0.025	**0.010**

Figure S1: Time alters rhizobial diversity and strain composition in the field soil control (FS). (A) Shannon diversity decreases after 2.5 years, while there are no significant differences in diversity between the three initial timepoints (See Table S5). (B) Time contributed to variation in the rhizobial population composition in soil (See Table S6).

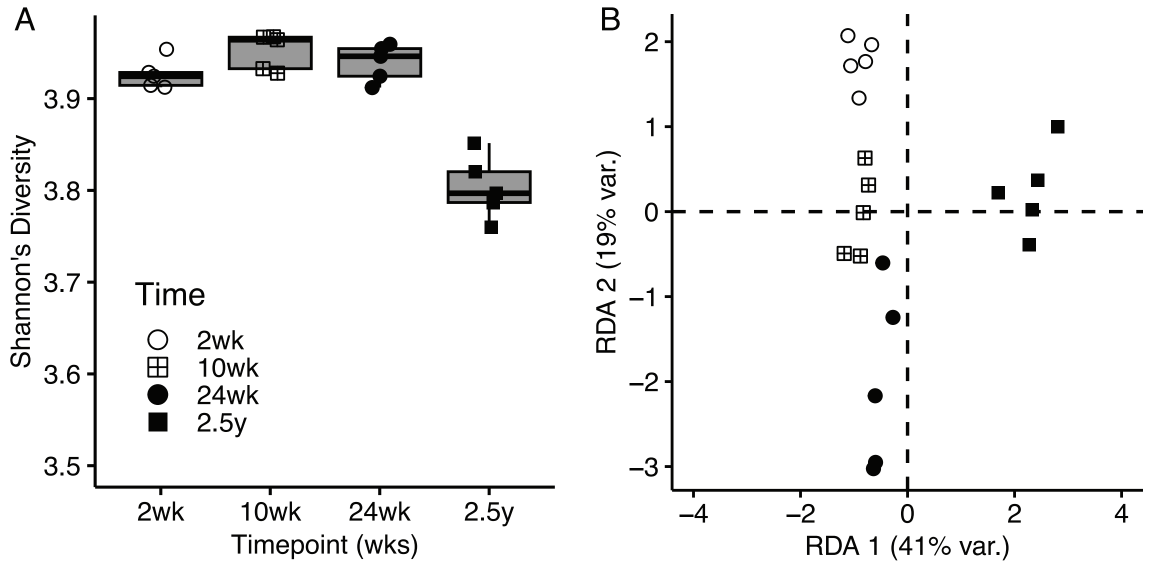

Figure S2: Strain relative fitness correlation coefficients (R) across all pairwise soil treatments at the 24-week timepoint are positive and significant. See Table S7 for R and *P* calculated for each Pearson correlation, and the color scale indicates the strength and direction of R.

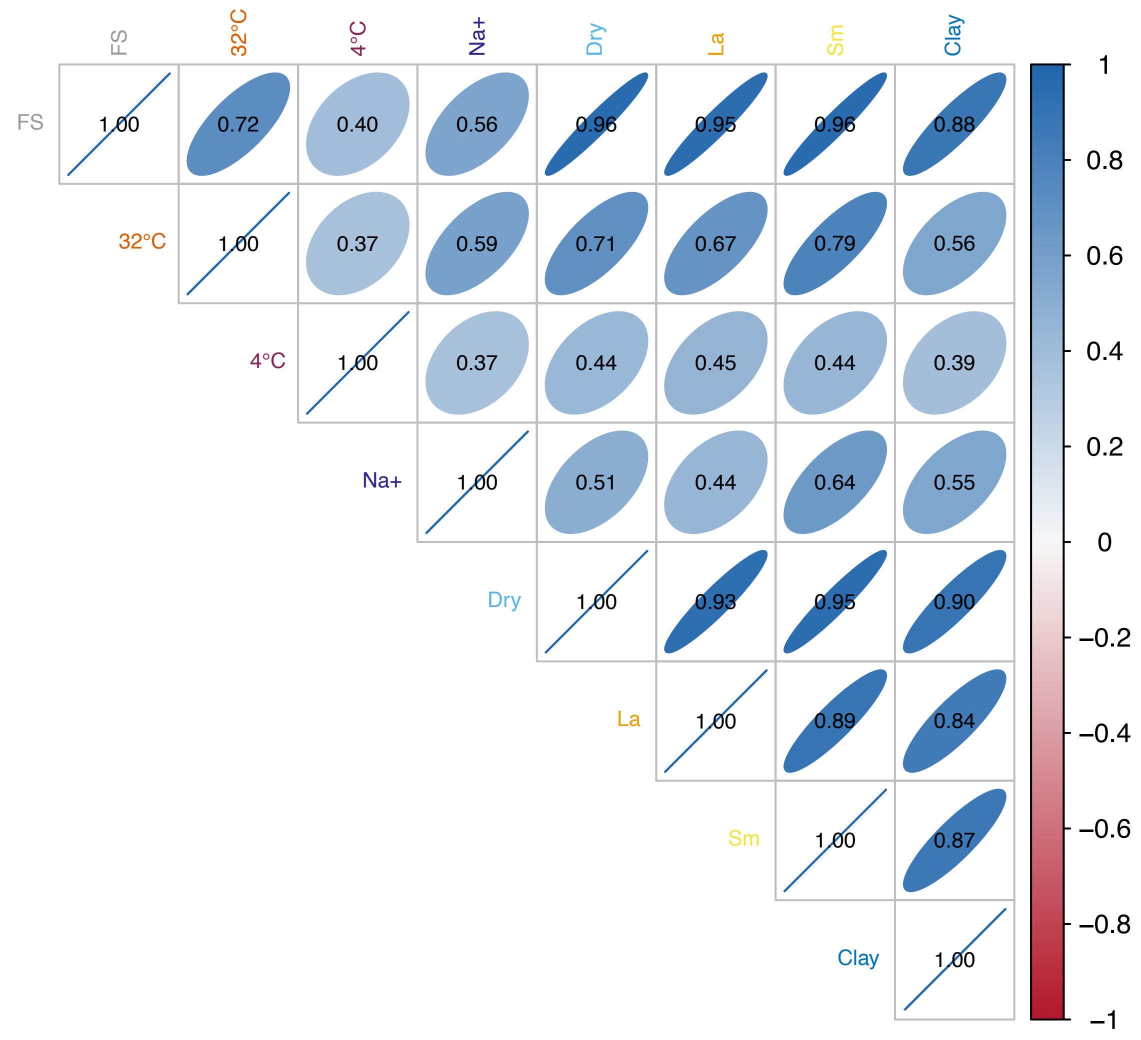

Figure S3: Rhizobial fitness in the soil is a complex trait with few LD groups showing statistical significance. Manhattan plot summarizing results of Genome-Wide Association (GWA) studies on rhizobial fitness in the seven soil environments tested. The x-axis shows the location of the LD group across the three replicons of the *Sinorhizobium meliloti* genome, and the y-axis shows the $-\log_{10}(P)$ of the strength of the association between strain fitness and genetic variation in each group. Colored dots indicate LD groups whose P-value (*P*) crossed the replicon-specific Bonferroni threshold (grey dashed lines). Significant associations were found at 4°C (purple circles), at 32°C (orange circles), or in clay (blue circles). See Table S9 for more information. The size of the circle was calculated by multiplying the absolute value of the allelic effect size by 10^5

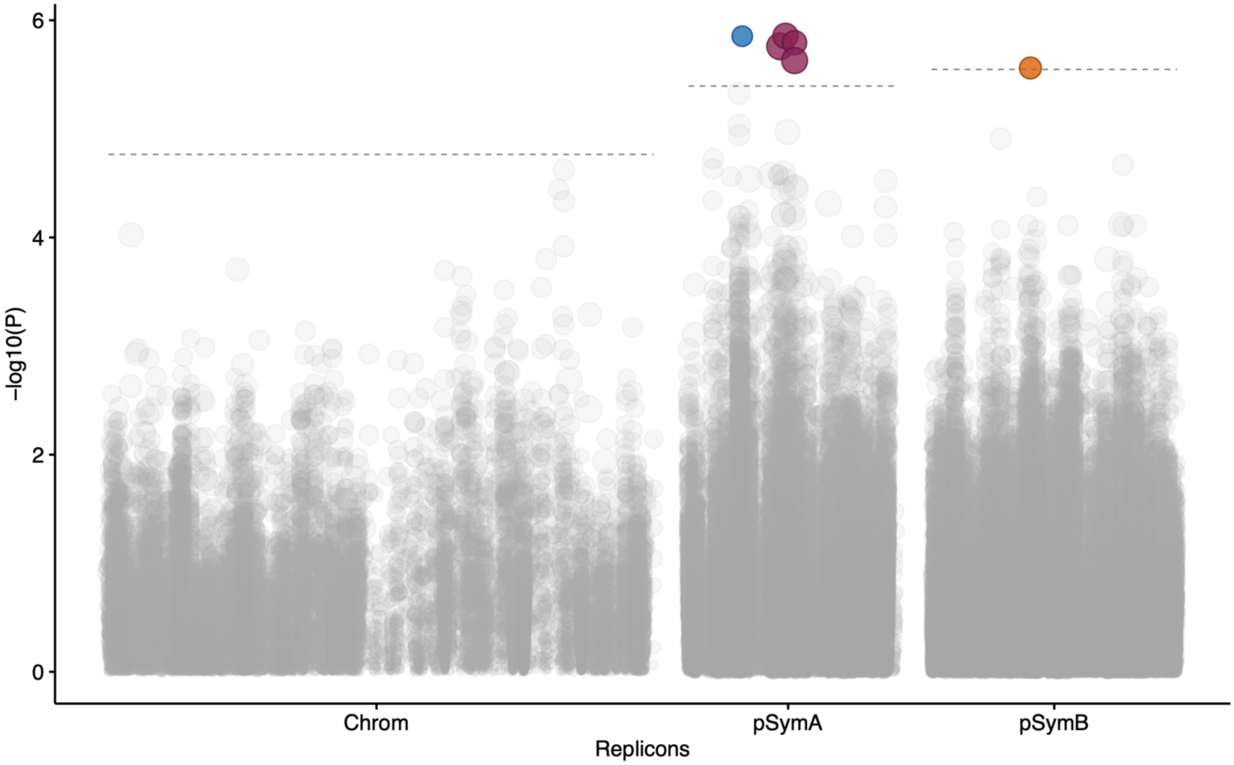

Figure S4: Pairwise comparisons between the allelic effect sizes (or beta values) of individual LD groups across all the soil conditions evaluated. Only 4°C (purple circle), 32°C (orange circle), and clay (blue circle) had LD groups that showed statistically significant association with rhizobia relative fitness (P-value crossed the Bonferroni threshold) in individual treatments. None of the LD groups were significant for more than one condition. The grey and transparent circles were not significant for any of the treatments.

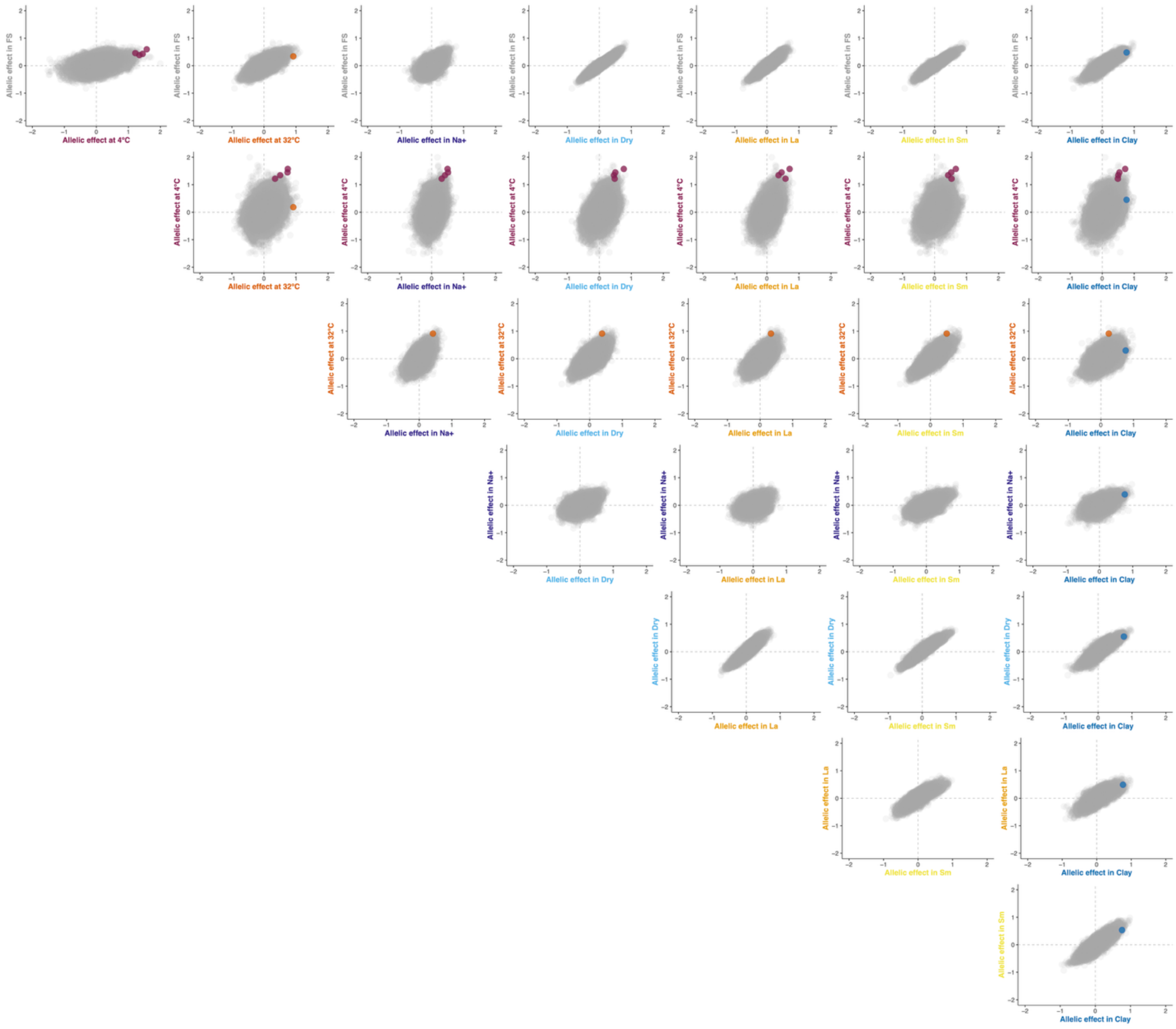

Figure S5: Pair-wise comparisons between the allelic effect sizes (or beta values) of individual LD groups between all the soil conditions and the two hosts, *Medicago truncatula* A17 and *Medicago* R108. The LD groups that show statistically significant association with rhizobia relative fitness (P-values cross the Bonferroni threshold) in either the host or the soil conditions are represented by circles that are either dark green (A17), light green (R108), purple (4°C), orange (32°C), and blue (Clay). None of the LD groups were significant for more than one condition. The grey and transparent circles were not significant for any of the treatments.

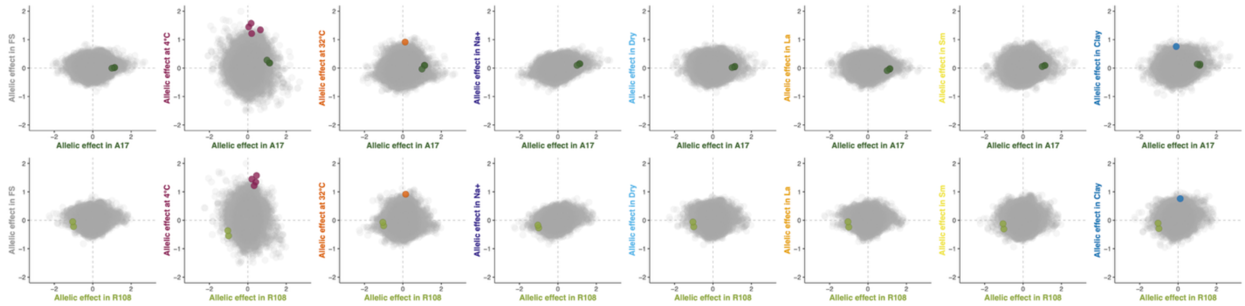

Figures S6: The rhizobial population selected by most soil treatments at 24 weeks has a higher or lower potential benefit than the initial inoculum, depending on the host plant, (A) *Medicago truncatula* A17, and (B) *Medicago littoralis* R108. We run a Dunnett's *post hoc* test comparing the potential benefit of the rhizobial population in the inoculum (Initial) vs the rhizobial population after each soil treatment. Samples are considered statistically significant when compared with the initial inoculum at: ***, $P < 0.001$; **, $P < 0.01$; *, $P < 0.05$. See Table S11B to see the Estimate, and P-value associated with this figure.

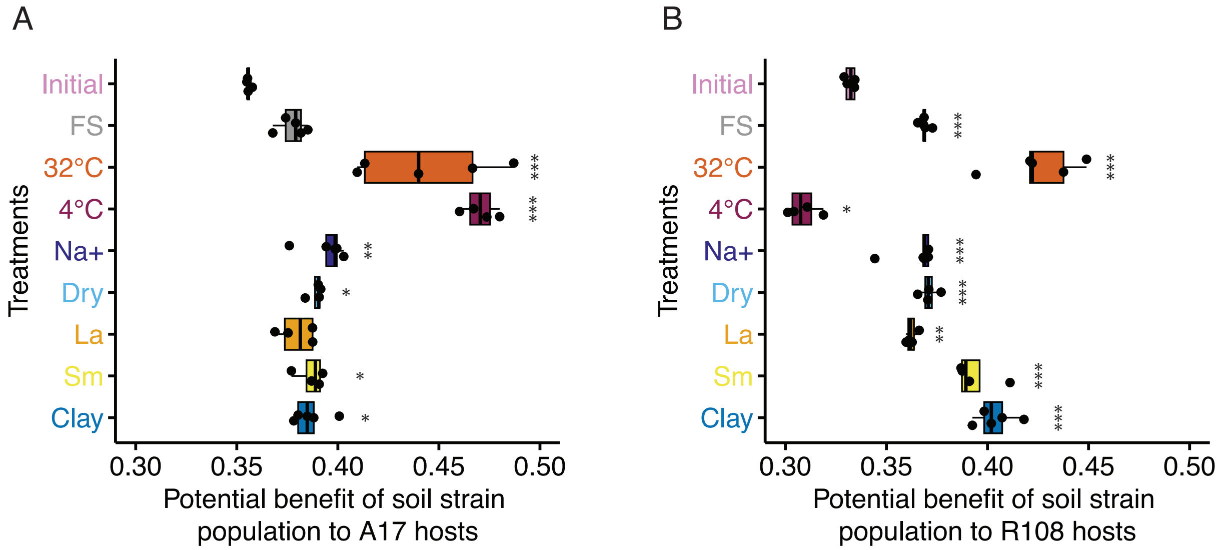

Supplement: Supplemental Material — Supplemental tables and figures. [file mbio.03793-25-s0001.pdf]
